# Longitudinal Changes in Health Utility Scores During the Perioperative Period Among Patients With Oral Cancer: An Analysis of the Effect of Older Age and Social Background

**DOI:** 10.1111/odi.70298

**Published:** 2026-03-15

**Authors:** Masashi Sasaki, Takayuki Aoki, Masahiro Uchibori, Masashi Tamura, Yasutaka Hoshimoto, Tsuyoshi Fukuzawa, Banri Tsuda, Yoshihide Ota

**Affiliations:** ^1^ Department of Oral and Maxillofacial Surgery Tokai University School of Medicine Isehara Kanagawa Japan; ^2^ Department of Radiation Oncology Tokai University School of Medicine Isehara Kanagawa Japan; ^3^ Department of Palliative Medicine Tokai University School of Medicine Isehara Kanagawa Japan

**Keywords:** EQ‐5D‐5L, health utility score, marital status, older adults, oral cancer, perioperative period

## Abstract

**Objective:**

Many countries have implemented health technology assessment of their public medical insurance programs owing to rising social security costs from ageing populations and expensive emerging medical technologies. Clarifying factors that affect health utility scores is essential for implementing treatments that reflect social values. Therefore, this prospective, observational study aimed to investigate the longitudinal changes in the health utility scores of patients with oral cancer eligible for radical treatment perioperatively.

**Methods:**

Overall, 219 patients with oral cancer who received standard therapy and completed utility assessments using the EQ‐5D‐5L at treatment completion and at 1, 3, 6 months, and 1 year post‐treatment were evaluated. Longitudinal changes in utility scores and factors influencing these scores were analysed.

**Results:**

The 1‐year post‐treatment utility scores were significantly higher than those before treatment (*p* < 0.01). Utility scores did not differ significantly between older and non‐older patients at all assessment time‐points. Marital status significantly influenced utility scores. Furthermore, utility scores did not differ significantly according to employment or living conditions.

**Conclusions:**

Health utility disadvantages are not associated with standard treatment for older patients with oral cancer, provided that they have good physical and cognitive functions. Additionally, considering social support for unmarried patients with cancer is vital.

## 1 Introduction

The cost of maintaining a social security system has increased in recent years owing to super‐ageing societies and the development of advanced but costly medical treatments (Aoki et al. 2019, 2022; Drummond et al. 2015; Longworth et al. 2014; Tosh et al. 2011). Furthermore, concerns have arisen about financial constraints on health insurance because of declining birth rates and slow economic growth. Therefore, many countries have implemented health technology assessment (HTA) of their public medical insurance programs to optimise healthcare costs (Drummond et al. 2015; Longworth et al. 2014; Tosh et al. 2011). In Japan, the government has passed a cabinet decision to introduce a cost‐effectiveness evaluation system into health insurance for medical care, with trial operations initiated in April 2016 (Basic Policies for Economic and Fiscal Management and Reform). In April 2019, HTA was implemented for expensive medicines and medical technologies.

HTA entails several methods, including cost‐utility analysis (CUA), cost‐effectiveness analysis, cost–benefit analysis and cost‐minimisation analysis (Tosh et al. 2011), with CUA being the most widely used (Tosh et al. 2011). CUA is performed using the incremental cost‐effectiveness ratio, which indicates the additional medical costs required to remain in perfect health for 1 year with new treatments and medical interventions (Detsky and Naglie 1990; Drummond et al. 2015; Longworth et al. 2014; Tosh et al. 2011). One year in perfect health is measured using the quality‐adjusted life year calculated from the health utility scores and survival time (Detsky and Naglie 1990; Drummond et al. 2015; Longworth et al. 2014; Tosh et al. 2011). Health utility scores are assessed using preference‐based measurement tools, such as the EuroQol‐5 Dimension (EQ‐5D) (Brooks 1996; EuroQol Group 1990; Herdman et al. 2011; Ikeda and Ikegami 1999; Ikeda et al. 2015; Kind et al. 1998; Luo et al. 2005; Nishimura et al. 1998; Sullivan et al. 2005; Shiroiwa et al. 2016, 2021; Tsuchiya et al. 2002; van Hout et al. 2012), Health Utilities Index (Feeny et al. 1995) and Short Form‐6 Dimension (Brazier et al. 1998), with the EQ‐5D being one of the most widely used instruments.

The older adult population is characterised by a decline in physical, cognitive and mental health, in addition to a high prevalence of significant comorbidities, inability to adapt to changing socio‐environmental conditions and financial problems (ESMO 2010; NCCN 2025; Kohn 1963; Ouchi et al. 2017). Japan's total population is decreasing, with a declining birth rate and an ageing population (Statistics Bureau n.d.). In October 2023, the proportion of the population aged ≥ 65 years and ≥ 75 years reached 29.1% and 19.1%, respectively (Statistics Bureau n.d.). Population ageing in Japan has also led to a rise in the incidence of oral and pharyngeal cancer. In 2019, oral and pharyngeal cancer was prevalent in 18.8 individuals per 100,000 population (Foundation for Promotion of Cancer Research 2023). Older patients with cancer may be ineligible for standard therapies because of frailty. However, radical treatment modalities in older patients provide survival benefits similar to those in younger patients (Saltzstein and Behling 2002; Shia et al. 2020). Cancer treatment in older patients is likely to be highly expensive owing to the greater incidence of complications caused by the decline in physical function and associated extended hospital stay. Furthermore, social isolation and loneliness have also been shown to affect utility scores (König and Hajek 2024). Marital status, employment status and household size may not only cause social isolation but also affect health utility scores. Value‐based medicine (VBM), an extension of evidence‐based medicine, is essential in medical care. The importance of quality of life (QOL), which reflects individual values and economic efficiency, which reflects social values, has been emphasised in medical care.

This study investigated the longitudinal changes in the health utility scores of patients with oral cancer who were eligible for radical treatment during the perioperative period, from the perspective of medical economics. In addition, it analysed the influence of older age and social backgrounds on the changes in health utility scores among patients with oral cancer.

## 2 Materials and Methods

### 2.1 Study Design and Population

This single‐cohort prospective observational study evaluated patients with oral malignant tumours who underwent radical treatment at the Department of Oral and Maxillofacial Surgery, Tokai University Hospital, Japan, between April 2018 and December 2023. The exclusion criteria were as follows: age < 20 years, multiple primary head and neck tumours, mental illness, inability to understand the questions or record their evaluations, missing utility values and refusal to participate. In addition, patients who experienced recurrence or metastasis during the follow‐up period, those who transferred to another hospital, patients who had missing data, loss to follow‐up, withdrawal of consent, or death from another disease were excluded. Figure 1 shows the patient selection flowchart.

**FIGURE 1:**
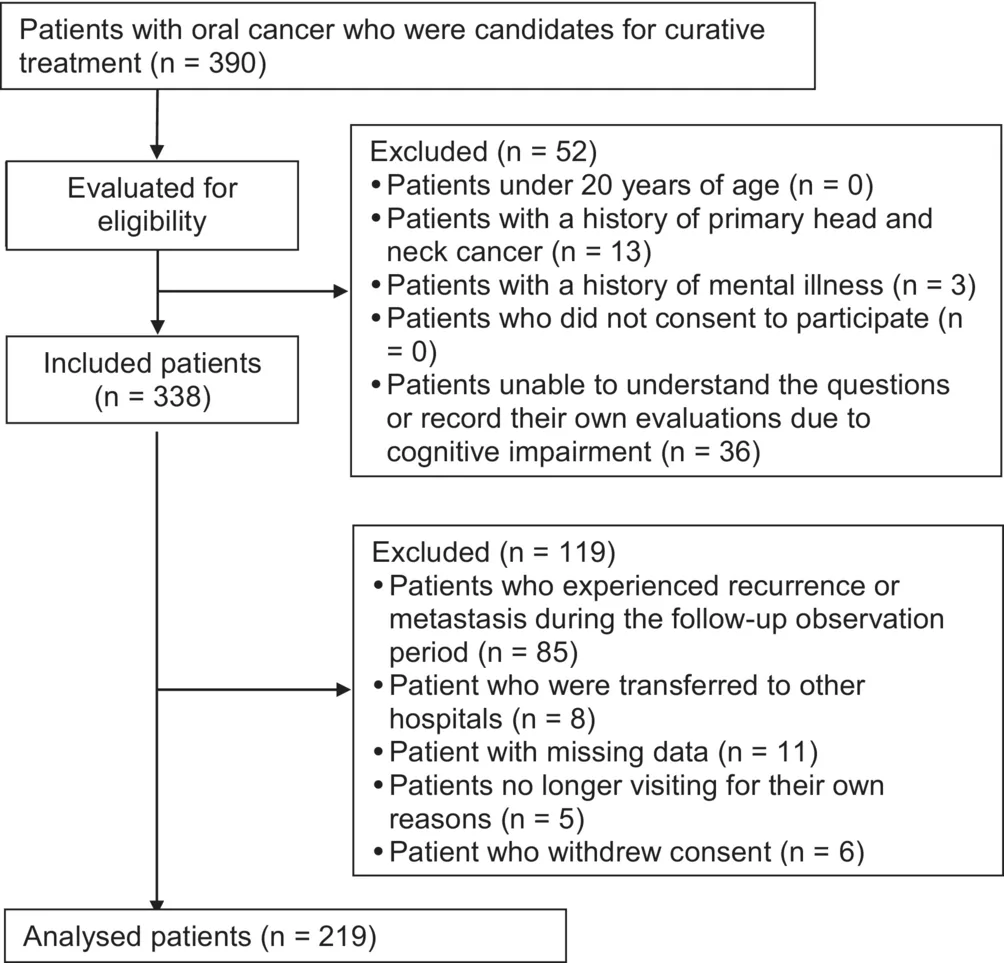
CONSORT flow diagram of the patient selection process.

### 2.2 Treatment Protocol

Patients were treated in accordance with the National Comprehensive Cancer Network (NCCN) Guidelines (Adelstein et al. 2017; Colevas et al. 2018; Pfister et al. 2015). Resectable cancer was treated using radical excision. Free‐flap reconstruction was performed for locally advanced malignant oral tumours. According to the NCCN guidelines, the features of oral squamous carcinoma with a high risk of recurrence include extranodal extension of lymph node metastasis and microscopic tumour‐involved resection margins based on histopathological diagnosis of the surgical specimen. In contrast, intermediate‐risk recurrence includes multiple lymph node metastases (Adelstein et al. 2017; Colevas et al. 2018; Pfister et al. 2015). Adjuvant radiotherapy (66 Gy/33 fractions) was recommended for these patients. Additionally, patients aged 75 years or younger with good performance status received chemoradiotherapy with tri‐weekly cisplatin (100 mg/m^2^) (66 Gy/33 fractions) (Figure 2).

**FIGURE 2:**
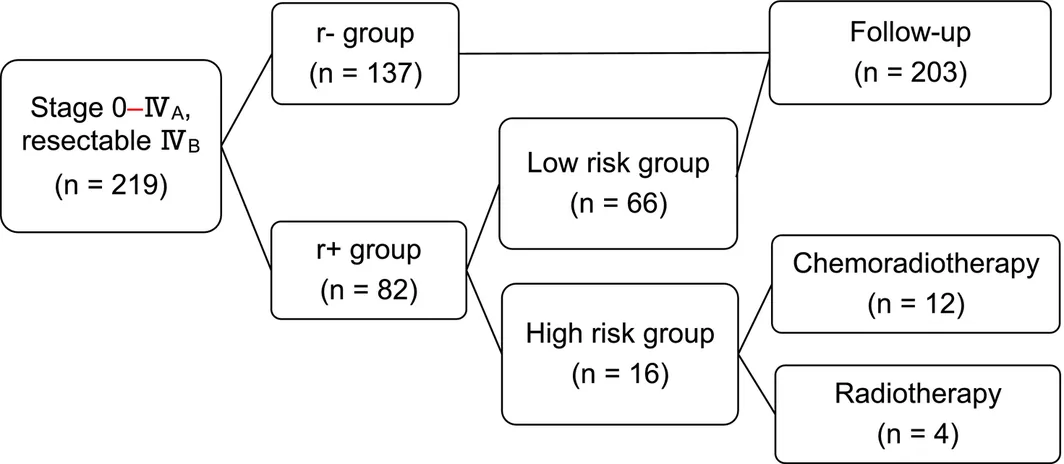
Treatment protocol. R group: Patients with early‐stage oral cancer who did not require reconstructive surgery; *r* + group: Patients with advanced oral cancer who required vascularised free‐flap reconstruction; high‐risk group: Patients with high‐risk characteristics, including multiple lymph node metastases, extranodal extension of lymph node metastasis, and microscopic tumour‐involved resection margins based on histopathological examination of the resected specimen.

### 2.3 Assessments

#### 2.3.1 Health Utility Scores

Health utility scores were assessed using the Japanese version of the paper‐based 5‐Level EuroQol‐5 Dimension (EQ‐5D‐5L) (Herdman et al. 2011; Ikeda et al. 2015; Shiroiwa et al. 2016, 2021; van Hout et al. 2012). Briefly, the EQ‐5D was developed by the EuroQol group (http://www.euroqol.org/) to assess health utility scores. It covers five health dimensions (domains): mobility, self‐care, usual activities, pain/discomfort and anxiety/depression (Brooks 1996; EuroQol Group 1990; Herdman et al. 2011; Ikeda and Ikegami 1999; Ikeda et al. 2015; Kind et al. 1998; Luo et al. 2005; Nishimura et al. 1998; Sullivan et al. 2005; Shiroiwa et al. 2016, 2021; Tsuchiya et al. 2002; van Hout et al. 2012). The EQ‐5D score is converted by the composite time trade‐off (cTTO) into a multi‐attribute utility index ranging from death (weighted 0) to perfect health (weighted 1) (Brooks 1996; EuroQol Group 1990; Herdman et al. 2011; Ikeda and Ikegami 1999; Ikeda et al. 2015; Kind et al. 1998; Luo et al. 2005; Nishimura et al. 1998; Sullivan et al. 2005; Shiroiwa et al. 2016, 2021; Tsuchiya et al. 2002; van Hout et al. 2012). The Japanese version of the EQ‐5D‐5L scoring system was developed based on the survey results of the general population categorised by sex and age at five sites throughout Japan (Brooks 1996; EuroQol Group 1990; Herdman et al. 2011; Ikeda and Ikegami 1999; Ikeda et al. 2015; Kind et al. 1998; Luo et al. 2005; Nishimura et al. 1998; Sullivan et al. 2005; Shiroiwa et al. 2016, 2021; Tsuchiya et al. 2002; van Hout et al. 2012). The health utility score (tariff) of the EQ‐5D‐5L was calculated using the EuroQol Group algorithm based on data from each respondent who was asked to evaluate 10 health states using the cTTO (Shiroiwa et al. 2016). In general, physical, mental and cognitive functions decline with ageing, and an individual's sense of value often changes. Therefore, the norm of utility value decreases as the individual ages (Shiroiwa et al. 2016, 2021).

Commonly, post‐operative symptoms subside within 1 year after surgery. Therefore, health utility scores were assessed before treatment, at treatment completion, and at 1, 3, 6 months, and 1 year after treatment. Data were collected by the attending physician or medical staff. The paper‐based EQ‐5D‐5L was directly handed to the patients, who then completed the form and returned it to the physician for aggregation and analysis. Most pre‐treatment data were collected on the day of admission. For patients receiving inpatient treatment, the treatment completion date was defined as the day of discharge. For patients who received post‐operative adjuvant therapy in the outpatient setting, the treatment completion day was defined as the day of treatment completion.

#### 2.3.2 Patient Characteristics

Many countries, including Japan, designate the older population as individuals whose chronological age is ≥ 65 years (Ouchi et al. 2017). However, recent studies have shown that currently, older individuals in Japan are more robust and active than their predecessors (Ouchi et al. 2017; Suzuki and Kwon 2006). Thus, a new definition of older adults as those aged ≥ 75 years has been proposed (Ouchi et al. 2017; Suzuki and Kwon 2006). Accordingly, the current study defined older and non‐older patients as those aged ≥ 75 years and < 75 years, respectively. The following patient characteristics were assessed: sex, age, primary cancer site, clinical stage (Union for International Cancer Control, TNM Classification of Malignant Tumours 8th edition), histological type, Eastern Cooperative Oncology Group Performance Status (ECOG PS) score, marital status (single or married), employment status (employed or unemployed), living condition (solitary or cohabiting), reconstructive surgery performed with a vascularised free flap, adjuvant therapy and treatment period (length of hospital stay) (Table 1). Treatment duration was defined as the length of hospital stay or the period from the day of ward admission to the day of completion of adjuvant therapy.

**TABLE 1:** Patient characteristics.

	All cases (*N* = 219)
Age	
Mean ± SE	65.16 ± 0.92
Range	21–90 (median: 68)
Older	≥ 75: 62, < 75: 157
Sex	
Male	122
Female	97
ECOG PS	
0	171
1	45
2	3
Social backgrounds	
Marital status	Single or Widowed: 55, Married: 164
Employment status	Unemployed: 118, Employed: 101
Living condition	Solitude: 22, Cohabitation: 197
Primary site	
Tongue	115
Mandibular gingiva	39
Buccal mucosa	17
Maxillary gingiva	15
Floor of the mouth	14
Mandible (intra‐osseous)	5
Palate	5
Sublingual gland	5
Submandibular gland	2
Lower lip	1
Subcutaneous	1
Clinical stage	
0	6
I	74
II	69
III	17
IVA	37
IVB	16
Histological types	
Squamous cell carcinoma	204
Salivary gland cancer	11
Malignant melanoma	1
Ameloblastic carcinoma	1
Odontogenic carcinoma	1
Sarcoma	1
Reconstructive surgery	
Performed	82
Not performed	137
Adjuvant therapy	16
Radiotherapy alone	4
Chemoradiotherapy	12

*Note:* Clinical stage is assessed according to the Union for International Cancer Control, TNM Classification of Malignant Tumours, 8th ed.; reconstructive surgery: reconstructive surgery with a vascularised free flap.

Abbreviation: ECOG PS, Eastern Cooperative Oncology Group Performance Status.

### 2.4 Statistical Analysis

Patient characteristics between the older and non‐older adult groups were compared using nonparametric chi‐squared tests. The Mann–Whitney U test was performed to analyse significant differences in the degree of tumour progression (clinical TNM stage), performance status and treatment duration between the two groups. Significant longitudinal changes in the EQ‐5D‐5L scores for each period were assessed using the Wilcoxon signed‐rank test. Significant differences in the EQ‐5D‐5L scores at each assessment time‐point between the older and non‐older adult groups, single and partnered groups, employed and unemployed groups and solitary and cohabiting groups were assessed using the Mann–Whitney U test. The ceiling effects of the EQ‐5D‐5L scores were determined by statistically analysing whether the sum of their respective mean and standard deviation scores exceeded their maximum scores. All statistical analyses were performed using SPSS 26 (IBM Corp., Armonk, NY). *p*‐values < 0.05 were considered significant.

## 3 Results

### 3.1 Patient Characteristics

Of the 390 patients initially enrolled, 219 patients were included in the final analysis (Figure 1). The median patient age was 68 years (range: 21–90 years, mean ± SE: 65.16 ± 0.92 years), and 62 patients were aged ≥ 75 years. A total of 164 patients had partners, 101 patients were employed and 22 patients lived alone (Table 1). All patients underwent radical surgical treatment. Overall, 82 patients underwent free‐flap reconstruction after wide resection for advanced cancer (*r* + group). Among them, 16 patients with a pathologically high risk of recurrence received adjuvant therapy (12 patients received chemoradiotherapy and 4 patients received radiotherapy alone) (Figure 2, Table 1).

### 3.2 Longitudinal Changes in the EQ‐5D‐5L Scores

The EQ‐5D‐5L scores were significantly higher 1 year after treatment than those before treatment (*p* = 0.000). The EQ‐5D‐5L scores were the lowest at treatment completion and gradually increased significantly thereafter (*p* < 0.01; Figure 3A). The EQ‐5D‐5L scores did not differ significantly between the older and non‐older adult groups at each assessment point. The EQ‐5D‐5L scores were the lowest at treatment completion in the non‐older adult group and 1 month after treatment in the older group (Figure 3B). In the non‐older adult group, the EQ‐ED‐5 L scores were significantly better at 1 year after treatment than those before treatment (*p* < 0.01; Figure 3B). The EQ‐5D‐5L scores at 3 and 6 months and 1 year post‐treatment were significantly higher among married and partnered patients than those among single and widowed patients (*p* < 0.01, p < 0.01, p < 0.01, respectively; Figure 3C). No significant differences in the EQ‐5D‐5L scores were observed between employed and unemployed patients (Figure 3D). With respect to living conditions, the EQ‐5D‐5L scores were higher in cohabiting patients than in those living alone, but the difference was only significant at 3 months after treatment (*p* < 0.01; Figure 3E).

**FIGURE 3:**
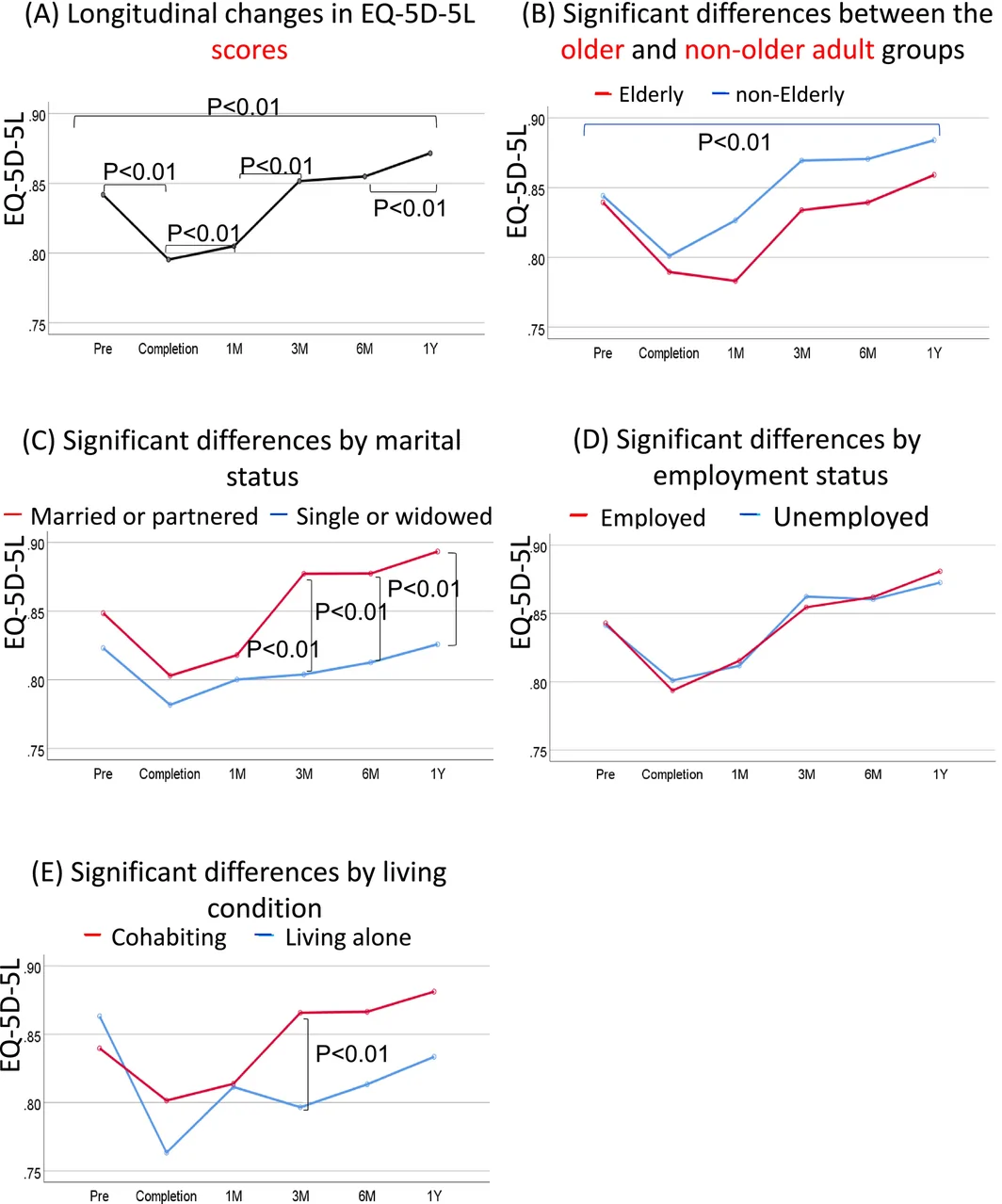
Longitudinal changes in the mean health utility scores during 1 year after treatment. (A) Longitudinal changes in the EQ‐5D‐5L. (B) Significant differences between the older and non‐older adult groups. (C) Significant differences by marital status. (D) Significant differences by employment status. (E) Significant differences by household size. Pre, before treatment; completion, at treatment completion; 1M, at 1 month post‐treatment; 3M, at 3 months post‐treatment; 6M, at 6 months post‐treatment; 1Y, at 1 year post‐treatment; EQ‐5D‐5L, 5‐Level EuroQol‐5 Dimension.

### 3.3 Effect of Older Age and Social Backgrounds on Health Utility

Sex, clinical stage, rate of reconstructive surgery, or treatment duration did not differ significantly between the older and non‐older adult groups; however, the ECOG PS scores were significantly higher in the older adult group (Table 2). Regarding social background, marital status, or living condition did not differ significantly between the two groups; however, significantly fewer older patients were employed (Table 2).

**TABLE 2:** Differences in patient characteristics between older and non‐older adult patients.

	Older adult group (*n* = 62)	Non‐older adult group (*n* = 157)	*p*
Sex			
Male	36	86	0.659
Female	26	71	
Age			
Median	79	62	
ECOG PS			
0	41	130	< 0.01*
1	19	26	
2	2	1	
Social background			
Marital status	Single: 18, Partnered: 44	Single: 37, Partnered: 120	0.401
Employment status	Unemployed: 53, Employed: 9	Unemployed: 65, Employed: 92	< 0.01*
Living condition	Solitude: 8, Cohabitation: 54	Solitude: 14, Cohabitation: 143	0.377
Clinical stage			
0	2	4	0.379
I	16	58	
II	23	48	
III	5	12	
IVA	13	24	
IVB	3	13	
Reconstructive surgery			
Performed	18	64	0.106
Not performed	44	93	
Treatment duration			
Mean ± SE	26.27 ± 3.41	31.26 ± 2.82	0.949

*Note:* Clinical stage is assessed according to the Union for International Cancer Control, TNM Classification of Malignant Tumours, 8th ed.; reconstructive surgery: reconstructive surgery with a vascularised free flap.

Abbreviation: ECOG PS, Eastern Cooperative Oncology Group Performance Status.

### 3.4 Ceiling Effect

The EQ‐5D‐5L did not show a ceiling effect in the study cohort (mean + SD = 0.842 + 0.128 = 0.970 < 1).

## 4 Discussion

In this study, no health utility disadvantages were observed in older patients who received radical treatment for oral cancer, provided that they had good physical and cognitive functions. In the non‐older adult group, the utility scores were significantly better at 1 year post‐treatment than at pre‐treatment. Meanwhile, no significant improvements were observed in the older adult group, and the changes resembled those seen in QOL. Collectively, these results imply that radical treatment can improve the health utility scores among non‐older patients with oral cancer, indicating that such treatment might be of crucial medical and economic value. Thus, our findings will be helpful in the establishment of oral cancer treatments that reflect VBM.

Ideally, oral cancer treatment should be based on the concept of VBM. The shorter life expectancy in older patients makes it difficult to determine the goals of cancer treatment. Older patients and their families should be closely consulted about focusing on extending survival time or maintaining the QOL. Aoki et al. (2021) analysed the perioperative QOL of patients with oral cancer who underwent curative treatment and found that the QOL was significantly lower in the older adult group (age ≥ 75 years) than in the non‐older adult group at 6 months post‐treatment (Aoki et al. 2021). Furthermore, the QOL of non‐older patients at 6 months post‐treatment was significantly better than that at pre‐treatment. However, no significant improvements were observed in older patients, with the scores even being lower than those at pre‐treatment (Aoki et al. 2021).

Utility scores, which reflect social values, are moderately correlated with the QOL (Aoki et al. 2019, 2022). In this study, the EQ‐5D‐5L instrument did not show a ceiling effect in patients with perioperative oral cancer. Aoki et al. (2022) reported that the EQ‐5D‐5L was correlated with the Functional Assessment of Cancer Therapy‐Head & Neck, a profile‐type QOL assessment tool, in patients with oral cancer (Aoki et al. 2022). In addition, they found that the EQ‐5D‐5L utility score was strongly represented in numerous factors for the physical well‐being and emotional well‐being (EWB) subscales in the FACT‐H&N, except for social/family well‐being (Aoki et al. 2022). Unusual activities, one of the five domains in EQ‐5D‐5L, corresponds to ‘work, study, housework, family or leisure activities’.

We investigated whether the utility scores of patients with oral cancer were truly unrelated to social background, such as employment status (employed or unemployed), marital status (single or married) and living condition (solitary or cohabiting). The results revealed significantly higher health utility scores in patients with oral cancer who had partners than in those who were single. König and Hajek (2024) investigated whether social isolation and loneliness were associated with utility scores using the EQ‐5D‐5L in a sample of 5000 German individuals aged between 18 and 74 years (König and Hajek 2024). They found that social isolation was associated with reduced health utility in individuals aged ≥ 40 years, whereas loneliness was associated with reduced health utility in all age groups. Furthermore, they reported that social isolation and loneliness were most strongly associated with the anxiety/depression domain of the EQ‐5D‐5L (König and Hajek 2024). This is consistent with the findings of previous studies, which showed that EWB, as assessed using the FACT‐H&N, influences the EQ‐5D‐5L scores (Aoki et al. 2022). This study demonstrated that social isolation, especially loneliness, could significantly affect health economics, necessitating the implementation of measures against loneliness and social isolation (König and Hajek 2024). Similarly, it revealed that marital status was an important factor influencing loneliness.

Several previous studies have shown that marital status influences the prognosis of patients with cancer (Aizer et al. 2013; Goodwin et al. 1987; Inverso et al. 2015; Li et al. 2023). For example, Goodwin et al. (1987) emphasized that the favourable effects of being married on overall survival are secondary to those of appropriate diagnosis, treatment and treatment response (Goodwin et al. 1987). Additionally, studies using Surveillance, Epidemiology and End Results data to examine the impact of marital status on cancer stage, use of definitive therapy and disease‐specific survival rates in patients with cancer, including those with head and neck cancer, in the United States showed similar findings (Aizer et al. 2013; Inverso et al. 2015; Li et al. 2023). Particularly, unmarried patients, including widows, were found to be at a significantly higher risk of metastatic cancer, undertreatment and cancer‐related death (Aizer et al. 2013; Inverso et al. 2015; Li et al. 2023). Furthermore, the social support provided by marriage had a consistent and significant effect on cancer detection, treatment and survival (Aizer et al. 2013; Inverso et al. 2015; Li et al. 2023).

If married individuals have worrying symptoms, their partners may encourage them to seek medical consultation and treatment (Aizer et al. 2013; Inverso et al. 2015). Additionally, married patients are more likely to adhere to prescribed treatments than unmarried patients (Aizer et al. 2013). Moreover, compared with unmarried patients, married patients experience less distress, depression and anxiety after a cancer diagnosis because their partners can share the emotional burden of the illness and provide adequate social support (Aizer et al. 2013; Inverso et al. 2015). This study shows that marital status affects health utility as evaluated using the EQ‐5D‐5L instrument, which includes the domains of usual activities and anxiety/depression. Social support interventions for vulnerable populations, including unmarried patients with cancer, may be a cost‐effective approach as they may improve outcomes and significantly increase health utility.

This study had some limitations. First, it was a single‐centre study. Second, many confounding factors may influence the relationship between marital status and utility score, such as educational background and annual income, which were not analysed in this study. Third, the study results were derived from patients with a good prognosis, without recurrence or metastasis. Fourth, the older adults included in this study were limited to those with good physical function who could undergo curative treatment. Furthermore, patients with cognitive impairment were excluded. Collecting utility data from older patients presents challenges.

Previous studies have shown that difficulty in responding to self‐rated health, a type of patient‐reported outcome, associated with cognitive decline in older individuals is a useful predictor of mortality (Mossey and Shapiro 1982; Pijls et al. 1993; Rakowski et al. 1994), highlighting the need for innovative approaches to HTA methodologies for these patients. However, despite these limitations, the results are robust and unlikely to be overturned.

In conclusion, perioperative health utility is not significantly different between older and non‐older patients with oral cancer receiving standard therapy. Therefore, radical treatments for older patients with oral cancer pose no disadvantages with respect to cost‐effectiveness, provided that these patients have good physical and cognitive functions. Marital status significantly affects health utility and QOL, including usual activities and anxiety/depression. Importantly, the most appropriate type of social support should also be considered in cancer management, especially for unmarried patients.

## Author Contributions

**Masashi Sasaki:** conceptualization, methodology, software, formal analysis, investigation, funding acquisition, writing – original draft, writing – review and editing. **Takayuki Aoki:** conceptualization, methodology, software, data curation, supervision, resources, project administration, formal analysis, validation, visualization, writing – review and editing, writing – original draft, investigation. **Masahiro Uchibori:** writing – review and editing, conceptualization, project administration, resources, investigation, data curation. **Masashi Tamura:** conceptualization, investigation, methodology, writing – review and editing, project administration, data curation. **Yasutaka Hoshimoto:** project administration, investigation, conceptualization, data curation, writing – review and editing, methodology. **Tsuyoshi Fukuzawa:** project administration, investigation, conceptualization, methodology, writing – review and editing, data curation, supervision. **Banri Tsuda:** project administration, conceptualization, methodology, writing – review and editing, investigation, data curation, supervision. **Yoshihide Ota:** conceptualization, methodology, writing – review and editing, investigation, data curation, project administration.

## Funding

This work was supported by the Japan Society for the Promotion of Science (JSPS) through a Grant‐in‐Aid for Scientific Research (C, KAKENHI) (Grant No. 23K09157).

## Ethics Statement

All procedures performed in this study were in accordance with the ethical standards of the institutional and/or national research committee and with the 1964 Helsinki Declaration and its later amendments or comparable ethical standards. The study was approved by the Institutional Review Board for Clinical Research of the Tokai University School of Medicine (15R‐11). To protect personal information, all the data were managed using an anonymous linking system.

## Consent

Informed consent was obtained from all participants included in the study.

## Conflicts of Interest

The authors declare no conflicts of interest.

## Data Availability

The data collected in the course of this study can be accessed from the corresponding author upon request.
